# Health Economics of Antibiotics

**DOI:** 10.3390/ph3051348

**Published:** 2010-04-29

**Authors:** Steven Simoens

**Affiliations:** Research Centre for Pharmaceutical Care and Pharmaco-economics, Katholieke Universiteit Leuven, O&N 2 P.O. Box 521, Herestraat 49, 3000 Leuven, Belgium; E-Mail: steven.simoens@pharm.kuleuven.be; Tel.: +32-16-323465; Fax: +32-16-323468

**Keywords:** health economics, antibiotics, cost-utility analysis, incremental cost-utility ratio

## Abstract

Antibiotics have made a significant contribution to improving patient health, but policy makers and health care payers are concerned about the costs of antibiotics in addition to their effectiveness. This paper aims to assess the value of antibiotics by examining incremental cost-utility ratios of antibiotics. Evidence was derived from cost-utility analyses of antibiotics included in the Tufts-New England Center Cost-Effectiveness Analysis Registry through September 2009. The analysis included 85 incremental cost-utility ratios from 23 cost-utility analyses. The findings showed that 38.8% of incremental cost-utility ratios related to dominant antibiotics (*i.e.,* more effective and less costly than the comparator); 45.9% referred to antibiotics that improved effectiveness, but also increased costs; and 15.3% related to dominated antibiotics (*i.e.,* less effective and more costly than the comparator). The median ratio was 748 € per quality-adjusted life year. Using threshold values of 20,000 € per quality-adjusted life year and 50,000 € per quality-adjusted life year, the probability that an antibiotic provides value for money was 64% and 67%, respectively. The current evidence base suggests that the majority of antibiotics provide value for money and that antibiotics can aid decision makers to attain further population health improvements, whilst containing pharmaceutical expenditures.

## 1. Introduction

Antibiotics have made a significant contributions to improving the health of patients suffering from bacterial infections. For instance, antibiotics are commonly used in the treatment of lower respiratory tract infections. The scientific literature and international guidelines recommend antibiotic therapy in patients with acute exacerbations of chronic obstructive pulmonary disease and community-acquired pneumonia [1,2,3]. Also, antibiotics appear effective in improving cure rates and decreasing duration of acute sinusitis in patients who have a microbiological diagnosis of bacterial infection or severe disease [4]. In fact, the added value of antibiotics for therapeutic and prophylactic purposes was so persuasive that many older antibiotics never underwent controlled clinical trials [5]. On the other hand, systematic reviews published by the Cochrane Collaboration have found no benefit of antibiotic therapy in upper respiratory tract infections because the cause of such infections is generally viral and, thus, the use of antibiotics is not indicated [6,7]. 

In an era of spiraling health care costs and limited resources, policy makers and health care payers are concerned about the costs of antibiotics, in addition to their effectiveness. Economic evaluation is a technique that can be used to determine whether antibiotic treatment adds sufficient value to justify its costs. An economic evaluation is defined as a comparative analysis of at least two health technologies in terms of both their costs and outcomes [8]. Evidence derived from economic evaluations is used to inform antibiotic pricing/reimbursement decisions in many countries. Antibiotics that provide better value are rewarded by means of a more favourable price/reimbursement. The requirement for economic evaluation fits within an overall trend towards evidence-based decision making in healthcare [9].

The results of an economic evaluation can be expressed in the form of an incremental cost-utility ratio (e.g. the additional cost per quality-adjusted life year (QALY) gained). Such a ratio relates the difference in costs between an antibiotic and the comparator to the difference in outcomes. The aim of this paper is to assess the value of antibiotics by examining incremental cost-utility ratios of antibiotics. Such information may serve to inform policy decisions relating to the use of antibiotics and to the allocation of scarce health care resources.

## 2. Methods

### 2.1. Cost-utility analysis

Evidence of the value of antibiotics was derived from cost-utility analyses. A cost-utility analysis measures costs and outcomes by means of specific health-related quality of life measures, such as QALYs. The QALY takes into account the quantity and quality of life. The quality of life associated with a health state is measured through the use of health utilities. A utility reflects the preference of an individual for the health state. Utilities are elicited on a scale of 0 (reflecting death) to 1 (reflecting perfect health). Quality of life data are then combined with estimates of the time period for which the outcomes last to generate QALYs.

Although cost-utility analyses represent a subset of economic evaluations, the study was limited to cost-utility analyses because this technique is recommended as the reference technique for economic evaluation in multiple countries [10], it provides an instrument to quantify the costs and outcomes of a medicine in a single ratio, it considers patient values in the measure of QALYs, it enables comparison of results across diseases, and it can inform decisions about how to allocate health care resources [8,11].

### 2.2. Data source

The Tufts-New England Center Cost-Effectiveness Analysis Registry was searched for original English-language cost-utility analyses of antibiotics [12]. The methodology underlying the Tufts-New England Center Cost-Effectiveness Analysis Registry is outlined in detail elsewhere [13]. Briefly, this Registry currently includes cost-utility analyses published from 1976 through September 2009. These cost-utility analyses were identified through an extensive search of MEDLINE using the keywords “quality-adjusted”, “QALY”, and “cost-utility analysis”. Two trained readers independently audit each cost-utility analysis and then convene to resolve any discrepancies. The data auditing form of each cost-utility analysis collects descriptive information about methods, incremental cost-utility ratio and utility weights.

### 2.3. Study variables

For each cost-utility analysis, the following variables were examined: publication year, target population, intervention type, country of patient sample, disease classification, prevention stage, funding source, study perspective, discounting, sensitivity analysis, incremental cost-utility ratio, and methodological quality.

Cost-utility analyses were included in the analysis if they studied an antibiotic intervention. The comparator could be another medicine, another health technology or no active therapy. Cost-utility analyses evaluating a combination of interventions (e.g. an antibiotic in combination with a medical device or a surgical procedure), were included if the comparison between intervention and comparator made it possible to single out the impact of the antibiotic.

As a cost-utility analysis may calculate separate incremental cost-utility ratios for patient samples belonging to different countries, information about the country of the patient sample rather than the study country was elicited. 

Cost-utility analyses related to all major disease areas. A distinction was made between three stages of prevention: primary prevention (measures preventing onset of condition), secondary prevention (interventions that identify and treat asymptomatic with risk factors or preclinical disease), and tertiary prevention (interventions limiting disability after harm has occurred). 

The source of funding of a cost-utility analysis was classified as “industry” (*i.e*. partial or complete funding by a pharmaceutical company), “non-industry” (e.g. government, foundation), “not specified” or “no funding”.

Cost-utility analyses were conducted from the perspective of society, the health care payer or an unspecified perspective.

In cost-utility analyses with a time horizon exceeding one year, time preference needs to be taken into account by means of discounting [14]. A categorical variable elicited whether discounting had been undertaken (“yes”), discounting had not been carried out (“no”) or it could not be determined whether discounting had been conducted (“not specified”). In cost-utility analyses with a shorter time horizon than one year, discounting was “not applicable”.

A sensitivity analysis can be carried out to determine the direction and the extent to which the results of an economic evaluation vary when estimates of input variables change [15]. A binary “yes/no” variable indicated whether a sensitivity analysis had been performed to explore the robustness of the incremental cost-utility ratio of antibiotics.

The analysis focused on cost-utility analyses that expressed results as an incremental cost per QALY gained. Ratios were actualized to 2008 values based on the evolution of the general consumer price index and were converted to Euro using exchange rates. This standardization of incremental cost-utility ratios made it possible to compare ratios between antibiotics and between disease areas. 

It should be noted that a cost-utility analysis may report multiple incremental cost-utility ratios pertaining to different antibiotics, comparators or patient populations, so that each cost-utility analysis may have contributed more than one ratio. Depending on the value of the antibiotic, the analysis reported that the antibiotic was dominant *(i.e.,* more effective and less costly than the comparator), the value of the incremental cost-utility ratio, or that the antibiotic was dominated (*i.e.,* less effective and more costly than the comparator).

An overall score of methodological quality was assigned to each cost-utility analysis on a Likert-type scale from 1 (lowest quality) to 7 (highest quality). A binary variable indicated whether evidence related to a “low-quality” cost-utility analysis with a quality score of 1.0–4.0 or a ‘high-quality’ cost-utility analysis with a quality score of 4.5–7.0.

### 2.4. Data analysis

Evidence of the value of antibiotics was summarized by calculating median incremental cost-utility ratios and frequency distributions. 

To explore associations between incremental cost-utility ratios and study characteristics, ratios were recoded into an ordinal variable consisting of 14 categories: more effective, less costly; ≤10,000 € per QALY; 10,001–20,000 € per QALY; 20,001–30,000 € per QALY; 30,001–40,000 € per QALY; 40,001–50,000 € per QALY; 50,001–60,000 € per QALY; 60,001–70,000 € per QALY; 70,001–80,000 € per QALY; 80,001–90,000 € per QALY; 90,001–100,000 € per QALY; 100,001–200,000 € per QALY; >200,000 € per QALY; less effective, more costly. Associations were examined by means of the Mann-Whitney U-test for ordinal variables. The statistical significance level was set at a p-value of 0.05. The analysis was carried out using SPSS Statistics 17.0.

## 3. Results

### 3.1. Search results

The search identified 34 articles. Articles were excluded for the following reasons: analysis of a combination of interventions without the possibility to single out the impact of an antibiotic (six articles) or analysis of an intervention that did not include an antibiotic (five articles). The analysis included 23 articles that reported information about 85 incremental cost-utility ratios of antibiotics [16,17,18,19,20,21,22,23,24,25,26,27,28,29,30,31,32,33,34,35,36,37,38]. The characteristics of included cost-utility analyses are described in Table 1. 

**Table 1:** Characteristics of cost-utility analyses of antibiotics.

Characteristic	Number (percentage)
*Publication year^a^*	
1985–2000	9 (39.2%)
2001–2005	7 (30.4%)
2006–2009	7 (30.4%)
*Country of patient sample^b^*	
Europe	11 (12.9%)
North America	57 (67.1%)
Other	17 (20)
*Disease classification^a^*	
Cardiovascular diseases	2 (8.7%)
Critical care	1 (4.3%)
Endocrine disorders	1 (4.3%)
Genito-urinary diseases	1 (4.3%)
Infectious diseases	13 (56.5%)
Musculoskeletal and rheumatologic diseases	1 (4.3%)
Respiratory diseases	3 (13.0%)
Sensory organ diseases	1 (4.3%)
*Prevention stage^a^*	
Primary	3 (13.0%)
Secondary	5 (21.7%)
Tertiary	15 (65.2%)
*Funding source^a^*	
Industry	3 (13.0%)
Non-industry	12 (52.2%)
No funding	1 (4.3%)
Not specified	7 (30.4%)
*Study perspective^a^*	
Society	9 (39.1%)
Health care payer	14 (60.9%)
*Discounting^a^*	
Yes	15 (65.2%)
Not applicable	8 (34.8%)
*Sensitivity analysis^a^*	
Yes	22 (95.7%)
No	1 (4.3%)
*Methodological quality^a^*	
1.0–3.5	5 (21.7%)
4.0–5.0	16 (69.6%)
5.5–7.0	2 (8.7%)

^a^ based on analysis of cost-utility analyses; ^b^ based on analysis of incremental cost-utility ratios.

The majority of original English-language cost-utility analyses of antibiotics have been published since 2000. The patient samples enrolled in these cost-utility analyses mainly originated from the United States and Canada (57%). Cost-utility analyses explored the cost-effectiveness of antibiotics for infectious diseases (56.5%) and respiratory diseases (13.0%). With respect to the prevention stage, the majority of studies related to tertiary prevention (65.2%), followed by secondary prevention (21.7%) and primary prevention (13.0%). The majority of cost-utility analyses of antibiotics received funding from sources other than industry (e.g. government, foundation) (52.2%). Thirteen percent of studies were funded by industry. Cost-utility analyses were generally carried out from the health care payer perspective (60.9%). Approximately 39% of studies took a societal perspective. When indicated, all studies discounted to take account of time preference. All but one cost-utility analysis undertook a sensitivity analysis. Around 70% of cost-utility analyses had a methodological quality score of 4.0–5.0. No study had a score exceeding 6.0.

### 3.2. Cost-utility

**Figure 1:**
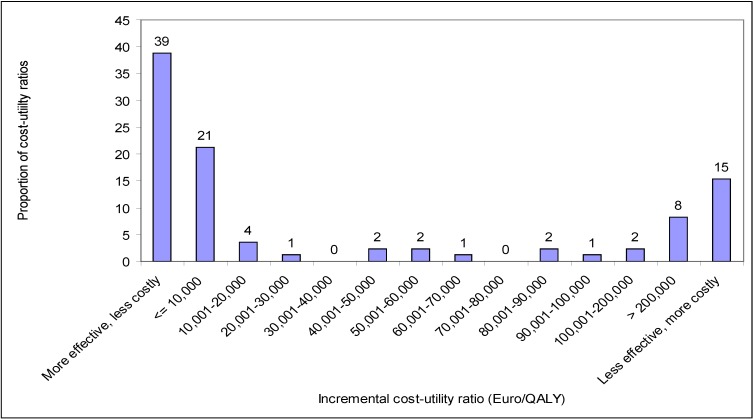
Frequency distribution of 85 incremental cost-utility ratios of antibiotics.

Figure 1 exhibits the frequency distribution of all 85 incremental cost-utility ratios of antibiotics. This Figure shows that 38.8% of incremental cost-utility ratios related to dominant antibiotics (*i.e.,* more effective and less costly than the comparator); 45.9% referred to antibiotics that improved effectiveness, but also increased costs; and 15.3% related to dominated antibiotics (*i.e.,* less effective and more costly than the comparator). The median incremental cost-utility ratio was 748 € per QALY (interquartile range: dominant—139,372 € per QALY). 

Considering all 85 incremental cost-utility ratios, it is possible to calculate the probability that an antibiotic provides value for money depending on a specific threshold value (see Table 2). This Table indicates that 64% of incremental cost-utility ratios were below or equal to 20,000 € per QALY; 67% of incremental cost-utility ratios were below or equal to 50,000 € per QALY; and 74% of incremental cost-utility ratios were below or equal to 100,000 € per QALY.

**Table 2:** Probability that an antibiotic provides value for money.

*Cost-utility*	*Probability*
More effective, less costly	39%
10,000 € per QALY	60%
20,000 € per QALY	64%
30,000 € per QALY	65%
40,000 € per QALY	65%
50,000 € per QALY	67%
60,000 € per QALY	70%
70,000 € per QALY	71%
80,000 € per QALY	71%
90,000 € per QALY	73%
100,000 € per QALY	74%
200,000 € per QALY	77%
>200,000 € per QALY	85%

The probability does not reach 100% because 15% of antibiotics were less effective and more costly than the comparator and, thus, cannot provide value for money irrespective of the threshold.

### 3.3. Cost-utility per disease

The median incremental cost-utility ratio was calculated per disease area. Focusing on diseases that were studied in at least 10% of cost-utility analyses, the median incremental cost-utility ratio was 748 € per QALY for infectious diseases (based on 49 ratios) and was dominant for respiratory diseases (based on 13 ratios).

### 3.4. Cost-utility per prevention stage

A comparison of median incremental cost-utility ratios of antibiotics indicated that tertiary prevention (median: dominant; based on 49 ratios) provides more value for money than secondary prevention (median: 241,352 € per QALY; based on 30 ratios). In turn, secondary prevention provides more value for money than primary prevention (median: 4,255,956 € per QALY; based on six ratios). However, no statistically significant difference was observed in the median ratio between preventive antibiotics (*i.e.,* primary prevention) and curative antibiotics (*i.e.,* secondary or tertiary prevention) (p = 0.119).

### 3.5. Cost-utility by funding source

The six incremental cost-utility ratios derived from analyses funded by industry tended to be lower (median: dominant) than the 45 incremental cost-utility ratios derived from analyses funded by non-industry (median: 102 € per QALY).

### 3.6. Cost-utility by study perspective

Comparing 32 incremental cost-utility ratios calculated from the societal perspective with 53 incremental cost-utility ratios calculated from the health care payer perspective, no statistically significant difference was observed in the median ratio between study perspectives (p = 0.285).

### 3.7. Cost-utility by methodological quality

Does methodological quality affect the size of incremental cost-utility ratios? To investigate this hypothesis, 27 incremental cost-utility ratios derived from ‘low-quality’ cost-utility analyses with a quality score of 1.0–4.0 were compared with 58 incremental cost-utility ratios derived from ‘high-quality’ cost-utility analyses with a quality score of 4.5–7.0. The results suggested that there were no statistically significant differences between ‘high-quality’ and ‘low-quality’ incremental cost-utility ratios (p = 0.146).

## 4. Discussion

This study has generated empirical evidence on the value of antibiotics by extracting 85 incremental cost-utility ratios from 23 cost-utility analyses. The database was diverse, covering various disease areas, prevention stages, target populations, antibiotics and comparators. This evidence suggested that the value of antibiotics covered the whole range from dominant antibiotics, antibiotics with positive incremental cost-utility ratios to dominated antibiotics.

Nevertheless, the majority of antibiotics provide value for money and some antibiotics even save money and improve effectiveness. Using threshold values of 20,000 € per QALY and 50,000 € per QALY, the probability that an antibiotic provides value for money was 64% and 67%, respectively. Examples of such antibiotics include empirical antibiotic treatment for adult patients with acute sinusitis symptoms, antibiotic-coated catheters for patients admitted to the intensive care unit, cotrimoxazole prophylaxis for children who are HIV/AIDS positive, cefaclor as second-line antibiotic treatment for children visiting family physicians’ offices and pediatric clinics with acute otitis media, and ciprofloxacin for outpatients with acute exacerbation of chronic bronchitis and a recent history of frequent exacerbations. Such medicines provide value as they raise effectiveness at a limited cost. Also, 38.8% of incremental cost-utility ratios related to dominant antibiotics. 

Focusing on cost-utility analyses that found evidence of dominated antibiotics, these analyses investigated the value of general strategies such as antibiotic prophylaxis or empirical antibiotic therapy rather than individual antibiotics. The use of dominated antibiotics in practice may originate from the fact that prescribers may not be aware of the health economic value of an antibiotic or the value may have deteriorated over time as a result of increasing resistance levels. 

Preventive measures using antibiotics do not necessarily provide value for money. Indeed, the data showed that primary preventive measures (*i.e.,* measures preventing onset of condition) include dominant antibiotics, antibiotics with a positive incremental cost-utility ratio and dominated antibiotics. This is corroborated by a recent study showing that the distribution of preventive measures using medicines in general spanned the full range of cost-utility results [39].

The majority of incremental cost-utility ratios of antibiotics originated from studies funded by sources other than industry. Although the limited number of observations made it impossible to test this hypothesis statistically, the analysis suggested that the value of antibiotics tended to be better in industry-funded studies than in studies funded by other sources. This observation may have several explanations: industry influences the design of economic evaluations with a view to improving cost-utility results; as R&D costs are high, industry markets those antibiotics that generate value for money only; industry sponsors economic evaluations of antibiotics that are likely to provide money only; researchers conduct and journal editors publish those economic evaluations that support the value of a antibiotics. In response to the possible manipulation of studies, professional societies and health care payers are increasingly issuing guidelines for the conduct and reporting of economic evaluations. 

In general, the value of antibiotics may be influenced by factors such as disease area, prevention stage, funding source, study perspective and methodological quality. Due to the small number of observations, it was not always possible to statistically test for associations between the value of antibiotics and a factor. Even when it was possible to apply a statistical test, the lack of significance may originate from the limited number of observations, rather than reflect the absence of an association. Future research needs to identify the factors that influence value by focusing on medicine classes for which more cost-utility analyses are published.

The results need to be interpreted with caution. Comparison of incremental cost-utility ratios is hindered by differences in the country, disease area, prevention stage, target population, antibiotic, comparator, funding source, study perspective and methodological quality of cost-utility analyses. Nevertheless, the results are similar to those observed in reviews of the cost-utility of medicines in general. Two literature studies have found that the majority of cost-utility analyses report favourable incremental cost-utility ratios for medicines and that industry-funded studies are more likely to report favourable incremental cost-utility ratios than non-industry funded studies [40,41]

This study suffered from several limitations. First, even though the study focused on cost-utility analyses included in a comprehensive registry from 1976 through September 2009, the number of observations was limited to 85 incremental cost-utility ratios from 23 cost-utility analyses. Second, the analysis may suffer from publication bias: only antibiotics that were evaluated and that earned publication in a peer-reviewed journal could be included in the sample. Nevertheless, the analysis included ‘negative’ results: 15.3% of incremental cost-utility ratios related to dominated antibiotics.

The objective of this study was to provide an aggregate analysis of the value of antibiotics. When interpreting the results, the reader needs to be aware that incremental cost-utility ratios are always selective and provide one piece of evidence only. Also, the number of incremental cost-utility ratios relating to a specific antibiotic may be limited. Furthermore, incremental cost-utility ratios are not static, thus underlining the importance of sensitivity analysis and the need for the reader to assess the transferability of results to his/her decision making context.

The value of antibiotics was derived from cost-utility analyses. As cost-utility analysis is only one technique of economic evaluation, this study does not provide a complete picture of the value of antibiotics. Evidence of the value of antibiotics was extracted from the Tufts-New England Center Cost-Effectiveness Analysis Registry. Although the database applies a comprehensive search strategy, some cost-utility analyses may have been missed and neither unpublished analyses nor analyses in a language other than English are included. Also, it should be noted that the trained readers who summarise and assess cost-utility analyses, are not blinded to the authors or journals in which studies are published, which may affect their judgement.

## 5. Conclusions

Health economic evidence about the value of antibiotics is available to decision makers to inform pricing/reimbursement decisions. The current evidence base suggests that the majority of antibiotics provide value for money and that antibiotics can aid decision makers to attain further population health improvements, whilst containing pharmaceutical expenditure.
